# Immunization of Experimental Dogs With Salivary Proteins From *Lutzomyia longipalpis*, Using DNA and Recombinant Canarypox Virus Induces Immune Responses Consistent With Protection Against *Leishmania infantum*

**DOI:** 10.3389/fimmu.2018.02558

**Published:** 2018-11-16

**Authors:** Melissa Moura Costa Abbehusen, Jurema Cunha, Martha Sena Suarez, Clarissa Teixeira, Valter dos Anjos Almeida, Laís da Silva Pereira, Marcelo Bordoni, Leonardo Gil-Santana, Manuela da Silva Solcà, Deborah Bittencourt Moté Fraga, Laurent Fischer, Patricia Torres Bozza, Patricia Sampaio Tavares Veras, Jesus G. Valenzuela, Shaden Kamhawi, Bruno B. Andrade, Claudia I. Brodskyn

**Affiliations:** ^1^Fundação Oswaldo Cruz, Instituto Gonçalo Moniz, Salvador, Brazil; ^2^Fiocruz Piauí, Fundação Oswaldo Cruz, Teresina, Brazil; ^3^Boerhinger Ingelheim, R&D, Laboratoire de Lyon Portes des Alpes, Lyon, France; ^4^Laboratório de Imunofarmacologia, Fundação Oswaldo Cruz, Instituto Oswaldo Cruz, Rio de Janeiro, Brazil; ^5^Vector Molecular Biology Unit, Laboratory of Malaria and Vector Research, National Institute of Allergy and Infectious Diseases, National Institutes of Health, Bethesda, MD, United States; ^6^Multinational Organization Network Sponsoring Translational and Epidemiological Research (MONSTER) Initiative, Fundação José Silveira, Salvador, Brazil; ^7^Escola Bahiana de Medicina e Saúde Pública, Salvador, Brazil; ^8^Universidade Salvador (UNIFACS), Laureate Universities, Salvador, Brazil; ^9^Faculdade de Medicina and Instituto de Ciências da Saúde, Universidade Federal da Bahia, Salvador, Brazil; ^10^Nacional de Ciência e Tecnologia de Investigação em Imunologia (III-INCT), São Paulo, Brazil

**Keywords:** vaccine, sand fly, canine visceral leishmaniasis, disease vectors, salivary proteins

## Abstract

Metacyclic *Leishmania* promastigotes are transmitted by sand flies that inject parasites and saliva into the host's skin. Previous studies have demonstrated that DNA plasmids encoding *Lutzomyia longipalpis* salivary proteins LJM17 and LJL143, when used to immunize dogs, resulted in a systemic and local Th1 cell-mediated immunity that interfered in parasite survival *in vitro*. Here we evaluated the ability of these same salivary antigens to induce anti-*Leishmania* immunity and to confer protection by immunizing dogs using a novel vaccination strategy more suitable for use in the field. The strategy consisted of a single dose of plasmid followed by two doses of recombinant *Canarypoxvirus* (*rCanarypoxvirus*) expressing *L. longipalpis* salivary proteins (LJM17 or LJL143). Thirty days after the final immunization, dogs were intradermally challenged with 10^7^ *Leishmania infantum* promastigotes in the presence of *L. longipalpis* saliva. We followed the experimentally infected dogs for 10 months to characterize clinical, parasitological, and immunological parameters. Upon vaccination, all immunized dogs presented strong and specific humoral responses with increased serum concentrations of IFN-γ, TNF, IL-7, and IL-15. The serum of dogs immunized with LJM17 also exhibited high levels of IL-2, IL-6, and IL-18. *L. infantum* infection was established in all experimental groups as evidenced by the presence of anti-*Leishmania* IgG, and by parasite detection in the spleen and skin. Dogs immunized with LJM17-based vaccines presented higher circulating levels of IFN-γ, IL-2, IL-6, IL-7, IL-15, IL-18, TNF, CXCL10, and GM-CSF post-infection when compared with controls. Results demonstrated that relevant *Leishmania*-specific immune responses were induced following vaccination of dogs with *L. longipalpis* salivary antigen LJM17 administered in a single priming dose of plasmid DNA, followed by two booster doses of recombinant Canarypox vector. Importantly, a significant increase in pro-inflammatory cytokines and chemokines known to be relevant for protection against leishmaniasis was evidenced after challenging LJM17-vaccinated dogs as compared to controls. Although similar results were observed following immunization with LJL143, the pro-inflammatory response observed after immunization was attenuated following infection. Collectively, these data suggest that the LJM17-based vaccine induced an immune profile consistent with the expected protective immunity against canine leishmaniosis. These results clearly support the need for further evaluation of the LJM17 antigen, using a heterologous prime-boost vaccination strategy against canine visceral leishmaniosis (CVL).

## Introduction

Visceral leishmaniasis (VL) is a severe, often lethal zoonosis caused by the intracellular protozoa *L. infantum*. This disease is endemic in the Mediterranean Basin, South America, and parts of Asia (1, 2). The presence of domestic dogs, considered the main domestic reservoir of the etiological agent of VL (*L. infantum*), in endemic areas is a known risk factor for human infection (3–5).

Parasite transmission to the vertebrate host occurs through the bite of infected sand flies during blood feeding (6). Parasites inoculation occurs in conjunction with sand fly saliva (7, 8) composed of pharmacologically potent molecules exerting anticoagulant, vasodilating, and anti-inflammatory activity, which can directly affect hemostasis and the inflammatory and immune response of the vertebrate host (9–11). A previous study by our group demonstrated that DNA plasmids encoding the *L. longipalpis* LJM19 salivary protein induced a strong DTH response in immunized hamsters that provided protection against lethal *L. infantum* infection. In addition, LJM19-immunized hamsters exhibited increased production of IFN-γ and IL-10 after exposure to uninfected sand fly bites, suggesting that this DTH response could be a marker of protection against infection by *L. infantum* (12).

Our collaborators identified two *L. longipalpis* salivary proteins(LJM17 and LJL143) that elicit DTH reactions in dogs subjected to repeated sand fly bites. These authors subsequently vaccinated dogs using these salivary proteins in a very complex immunization strategy involving three intramuscular injections of DNA plasmids codifying LJM17 or LJL143, followed by one dose by intradermal route and two subsequent doses by intramuscular route coupled to electroporation of the same antigens. They then intradermally injected the two antigens as purified recombinant salivary proteins associated with CpG, and finally boosted with recombinant *Canarypoxvirus* expressing LJL143 or LJM17. The intention of these investigators was to elicit a strong response against the two proteins in an attempt to determine whether the generated immune response was capable of protecting the animals from *L. infantum* infection. These authors observed that LJM17 immunization induced enhanced RNA synthesis of IL-12, while the LJL143 protein provoked a mixed response as evidenced by the expression of IL-12 and IL-4 at the vaccine inoculation site. Moreover, in an *in vitro* killing assay, dogs immunized with LJM17 or LJL143 presented a reduction in the number of infected macrophages in the presence of autologous lymphocytes (13).

Due to the promising nature of these results, we chose to adapt this immunization strategy to formulate a simpler version more compatible with future potential use in the field, consisting of three intramuscular doses. Therefore, a single dose of plasmids encoding the two *L. longipalpis* salivary proteins (LJM17 or LJL143) was followed by two booster doses of *rCanarypoxviruses* vector expressing one of the two aforementioned *L. longipalpis* salivary protein genes. In addition to evaluating the immune response developed by immunized dogs, we also analyzed immunological, parasitological, and clinical parameters during the follow up of the dogs corresponding to 10 months after experimental infection with *L. infantum* in the presence of *L. longipalpis* saliva.

## Materials and methods

### Animals

Thirty beagle dogs of both sexes, aged 2 to 3 months, were acquired in Paraná, Brazil. Throughout the study, these animals were housed at the Experimentation Kennel facility in the region of Monte Gordo, located in the municipality of Camaçari, Bahia-Brazil. All dogs received routine vaccinations (rabies, distemper, hepatitis/*Adenovirus* type2, leptospirosis, *Parvovirus* and *Coronavirus*) and were dewormed. Blood samples were collected for serological evaluation and no dogs showed any detectable levels of antibodies against *Leishmania, L. longipalpis* saliva, *Ehrlichia canis, Borrelia burgdorferi*, or *Dirofilaria immitis*. Dogs were kept under surveillance and received veterinary medical care, a balanced feed, water *ad libitum*, and were housed in covered kennels with stalls protected by thin netting to prevent natural exposure to vectors, with animals grouped up to five per stall, separated according to gender. These procedures were kept during for all the period of the study (immunization, infection, and following up).

### Ethics statement

All procedures were conducted according to the guidelines for animal research of the Brazilian College of Animal Experimentation (Colégio Brasileiro de Experimentação Animal) and the National Council of Animal Experimentation Control (Conselho Nacional de Controle de Experimentação Animal). The IGM—FIOCRUZ Institutional Review Board for Animal Experimentation approved all procedures (CEUA—Instituto Gonçalo Muniz—IGM/FIOCRUZ—protocol number: 020/2011). At the end of the study all animals were euthanized. The dogs were sedated with acepromazine (0.1 mg/kg, iv, Acepram 1%, Vetnil, Brazil) and sodium thiopental (15 mg/kg, iv, Thiopentax 1 g, Cristália Brazil) and euthanized using a saturated solution of potassium chloride (2 mL/kg, iv).

### Immunization strategies

We performed a randomized placebo-controlled study using 30 beagle dogs randomly divided into two treatment groups, each with a different formulation of anti-*Leishmania* vaccine (LJM17 and LJL143), as well as a control group (saline). Each group consisted of 10 dogs. An initial immunization was performed using 250 μg of plasmid DNA encoding the *L. longipalpis* salivary protein LJM17 (pNBO002) or LJL143 (pNBO003), injected intramuscularly. After an interval of 28 days, a second immunization containing 10^8^ *recombinant Canarypoxvirus* expressing the gene encoding LJL143 proteins (vCP2389) or LJM17 (vCP2390) was also administered by intramuscular route, followed by a third and final inoculation 42 days after the first immunization. The control group received an identical volume of saline (Supplementary Figure 1). After each immunization, the occurrence of adverse reactions was evaluated by clinical examination, with any systemic or local signs (localized pain, itching, swelling, lumps, fever, etc.) recorded.

### Sand flies and sonicate gland homogenate (SGH) preparation

*L. longipalpis*, Cavunge strain (Cavunge, Bahia), were reared at the Laboratory of Imunoparasitology, IGM-FIOCRUZ, Bahia-Brazil, as previously described (12). Salivary glands were dissected from 5 to 7 day-old females and stored in saline at −70°C. Before use, salivary glands were sonicated and centrifuged at 8,000 x g for 5 min. The supernatant was collected and used immediately.

### *Leishmania* parasites and intradermal experimental infection

The experimental infection challenge employed a previously described protocol (14). Briefly, *L. infantum* (MCAN/BR/00/BA262) promastigotes isolated from a naturally infected dog (Bahia State, Brazil) were cultured in Schneider's medium (LGC, Brazil) supplemented with 10% heat-inactivated FBS (fetal bovine serum), 2 mM L-glutamine, 100 IU/ml penicillin and 1% streptomycin. The infectivity level of these parasites was tested in hamsters prior to performing infection procedures in dogs, which were proven to cause severe disease in this animal model. Dogs were inoculated intradermally 30 days after the final immunization using a 29-gauge needle at a volume of 200 μl in the right ear with 10^7^ stationary-phase promastigotes in the presence of Sonicated Salivary Gland Homogenate (SGH) equivalent to five pairs of glands. After the infection challenge, all dogs were again housed in a kennel protected by anti-insect netting.

### Assessment of humoral immune response

Anti-LJM17 and LJL143 IgG levels were measured both prior to the initial immunization and 15 days after the third immunization (Time zero = T0). Anti–LJM17 and LJL143 IgG was detected by ELISA as previously described (13).

Levels of anti-*Leishmania* IgG antibodies were measured every 2 months until 10 months after infection by (ELISA) using Soluble *Leishmania* antigen (SLA) as described previously (14).

### Cytokine and chemokine detection by luminex assay

A bead-based multiplex assay employing Luminex technology (Milliplex Map Kit - canine cytokine magnetic bead panel, Life Technologies, Carlsbad, CA, USA) was used to measure a variety of cytokines and chemokines (IFN-γ, IL-10, TNF, IL-1β, IL-2, IL-6, IL-7, IL-15, IL-8, MCP-1, CXCL-1, GM-CSF) in canine sera. This kit was used in accordance with the manufacturer protocol, as previously described (15). We measured cytokines and chemokines in the serum of immunized (T0) and infected dogs 2 and 4 months after infection (T1 and T2). The present assay was performed at the Laboratory of Immunopharmacology of the Oswaldo Cruz Institute, Fiocruz – RJ.

### ELISA evaluation of IFN-γ and IL-10 production by peripheral blood mononuclear cells (PBMCs) stimulated with salivary proteins or *leishmania*

At T0, IFN-γ, and IL-10 production was assessed by stimulation with LJM17 or LJL143 in the PBMCs of immunized and control dogs as previously described (13). Similarly, every 2 months after infection, cytokine production was again quantified following stimulation with *L. infantum* as previously described (14).

### Clinical evaluation

After challenge, all dogs underwent a blind clinical evaluation every 2 months until 10 months after infection. CVL severity was defined based on the presence or absence of the following clinical signs: nutritional status, as represented by weight loss, skin involvement, lymphadenomegaly, conjunctivitis, toenail size (onychogryphosis) and splenomegaly, graded from 0 to 2 at each time point, adapted from Manna et al. (16). After the final evaluation, the total of these values was considered as the clinical score for each dog (minimum score zero, maximum 16).

### DNA extraction and parasite burden quantification by real-time PCR

At T1 and T2 (2 and 4 months after infection, respectively), ear skin biopsies and aspiration punctures from bone marrow, popliteal lymph nodes, and spleens were collected as previously described (17, 18). Real Time PCR was performed to quantify parasite load as previously described (14). In addition, we used part of the splenic samples to culture the parasites in NNN biphasic medium (Novy-MCNeal-Nicolle) supplemented with 20% Fetal Bovine Serum and 100 μl of gentamicin to avoid contamination as described by Barrouin-Melo et al. (17).

### Statistical analysis

Statistical analysis was performed using GraphPad Prism v5.0 (GraphPad Software, USA). Group comparisons were made considering levels of antibodies, parasite load, and cytokines using the non-parametric Mann Whitney *U*-test. Hierarchical cluster analysis (Ward's method) with bootstrap was performed to depict the overall expression profile of serum biomarkers in the negative and experimentally infected dogs. Significant statistical differences between more than two groups were evaluated using the Kruskal-Wallis test with Dunn's multiple comparisons. Differences were considered significant when *P*-values ≤ 0.05.

## Results

### Dogs immunized with LJM17 and LJL143 salivary proteins presented a mixed immune response

**Figure 1:**
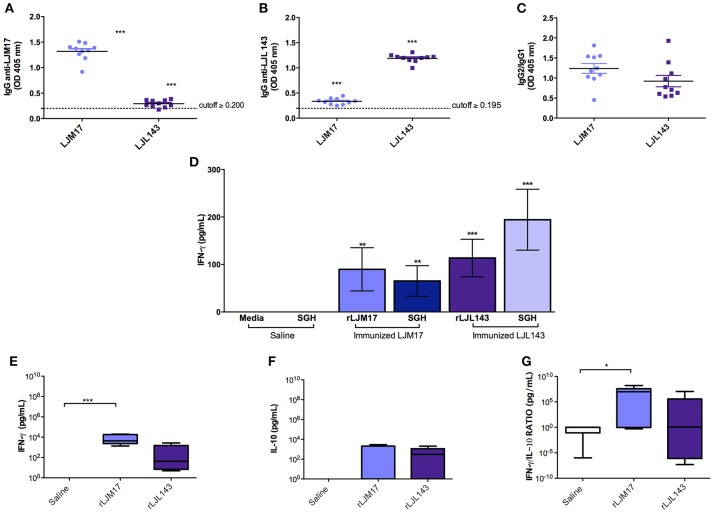
Analysis of humoral and cellular immune response in immunized and control dogs. Beagles (n = 10 animals/group, total of 30 dogs) were immunized as described in the methodology section. Sera collected at T0 (15 days after the last immunization) were used to detect total IgG **(A)** anti-LJM17 or **(B)** anti-LJL143 and **(C)** specific IgG1/IgG2 subclasses. Dotted lines represent cutoffs obtained from sera of healthy dogs prior to immunization. PBMCs of immunized and control animals were stimulated with 4 μg/mL of rLJM17 or rLJL143, and/or SGH of *L. longipalpis*, and supernatants were collected after 48 h, then analyzed by ELISA to detect IFN-γ **(D)**. Luminex for the detection of IFN-γ **(E)**, IL-10 **(F)** and IFN-γ/IL-10 **(G)**. Statistical analysis was performed using the Kruskall-Wallis test with Dunns post-test (*p < 0.05, **p < 0.005, ***p < 0.0001).

The occurrence of adverse reactions due to immunization schedule was not observed. ELISA was performed to detect anti-LJM17 and LJL143 IgG, IgG1, and IgG2 subclasses in the sera of immunized and control dogs at baseline and T0 (15 days after the last immunization). The immunized groups showed significant production of specific antibodies against the two proteins (*P* < 0.0001) when compared to controls (Figure 1A,B; Supplementary Table 1), demonstrating the immunogenicity of these salivary gland proteins. Our analysis of the anti-LJM17 and LJL143 IgG1 and IgG2 subclasses revealed humoral responses against the corresponding proteins, with enhanced IgG2 response (Figure 1C; Supplementary Table 1).

Cytokine production was evaluated by ELISA following stimulation with a recombinant protein (LJM17 or LJL143), *L. longipalpis* SGH (equivalent to 1 pair/mL) or Concanavalin A as a positive control. PBMCs from immunized dogs stimulated with SGH or one of the recombinant proteins exhibited higher IFN-γ production compared to controls. (*P* < 0.005) (Figure 1D; Supplementary Table 2). In addition, no IL-10 production was observed in any of the experimental groups, which suggests that a Th1 response was induced by immunization with the salivary proteins (Data not shown).

Our evaluation of circulating cytokines in the sera of experimental and control groups after immunization found that the groups immunized with salivary proteins presented higher levels of IFN-γ and IL-10 (Figures 1E,F; Supplementary Table 2). A pro-inflammatory response profile was observed, as evidenced by significantly higher IFN-γ production in the LJM17-immunized group (*P* < 0.0002) (Figure 1E; Supplementary Table 2), confirmed by the elevated IFN-γ:IL-10 ratio in comparison to the control group (*P* < 0.0254) (Figure 1G;Supplementary Table 2).

### IgG anti-*leishmania*, parasite load quantification, and clinical evaluation after infection

**Figure 2:**
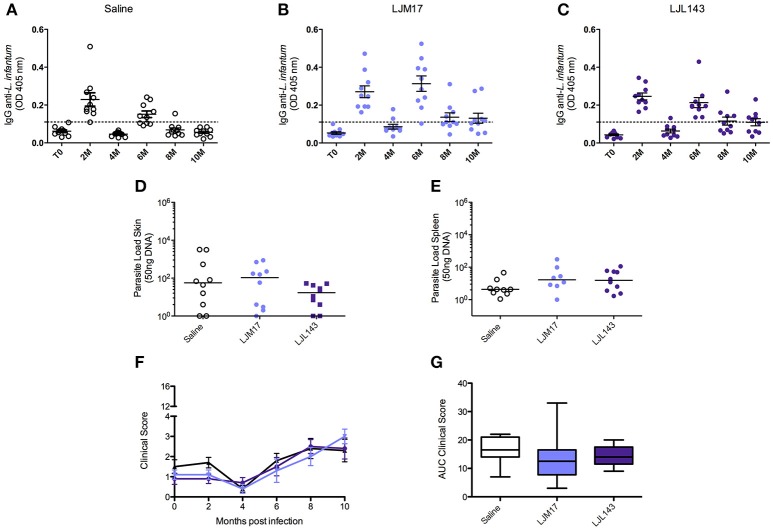
Analysis of humoral immune response in canine sera, parasite loads and clinicial evaluation after challenge. Beagle dogs (*n* = 10 animals/group; total of 30 dogs) were immunized following vaccination strategies described in the methodology section. Thirty days after the final immunization, all dogs were challenged with *L. infantum* in the presence of *L. longipalpis* saliva. Sera from the immunized and control animals were collected at T0 (15 days after immunization),and every 2 months until 10 months after infection, to detect anti-*Leishmania* IgG antibodies. **(A)** Control, LJM17 **(B)**, and LJL143 **(C)** immunized dogs T0 to 10 months after infection. The dotted line represents the cutoff point (mean plus 2x standard deviation = 0.036) obtained from sera of healthy dogs prior to infection. Samples of skin **(D)** and spleen **(E)** from immunized and control animals were collected at 2 months post infection for parasite load quantification by real time PCR. Clinical evaluation score curves **(F)** and Area Under Curve (AUC) **(G)** from T0 to 10 months after infection. Statistical analysis was performed using the Kruskall-Wallis test with Dunns post-test.

To evaluate the humoral immune response induced by infection under the experimental protocol employing *L. infantum* promastigotes and *L. longipalpis* saliva gland homogenate, an ELISA assay was performed to detect anti-*Leishmania* IgG in canine sera at zero (T0), and every 2 months after the infection. At 2 months after infection, anti-*Leishmania* antibodies had already been detected in all animals. The groups that received LJM17 presented significantly higher levels of IgG in comparison to controls (*P* < 0.0001). However, at 4 months after infection antibody concentrations decreased and all animals exhibited negative serology. Interestingly, 6 months after infection, we found an increase in the serological levels of anti-*Leishmania* antibodies (Figures 2A–C; Supplementary Table 3), yet no parasites were detected in the skin, spleen, bone marrow or lymph nodes at this time point (Data not shown).

In order to evaluate the protective ability conferred by the experimental immunization protocol, parasite load was assessed by Real Time PCR during the 10 months of follow up. However, only at 2 months after infection (T1), parasites were detected only in the skin and in the spleen (Figures 2D,E; Supplementary Table 4) in both immunized and control groups. After this time point, no parasites were detected in any biological samples. The parasites quantified in the qPCR were viable, because we re-isolated the parasites from splenic samples of experimental and controls groups by culturing them in specific medium.

However, the absence of statistical significance among the evaluated groups indicates that although the dogs were effectively infected, albeit very mildly, we are unable to confirm any preventive action with respect to the immunization protocol.

After the infection, during the clinical evaluation period (10 months), no specific signs of CVL and, consequently, no significant increases in clinical scores were observed in any of the dogs in either the control or immunized groups (Figures 2F,G;Supplementary Table 5).

### LJM17-immunized dogs present elevated IFN-γ in *L. infantum-*stimulated PBMCs and sera

**Figure 3:**
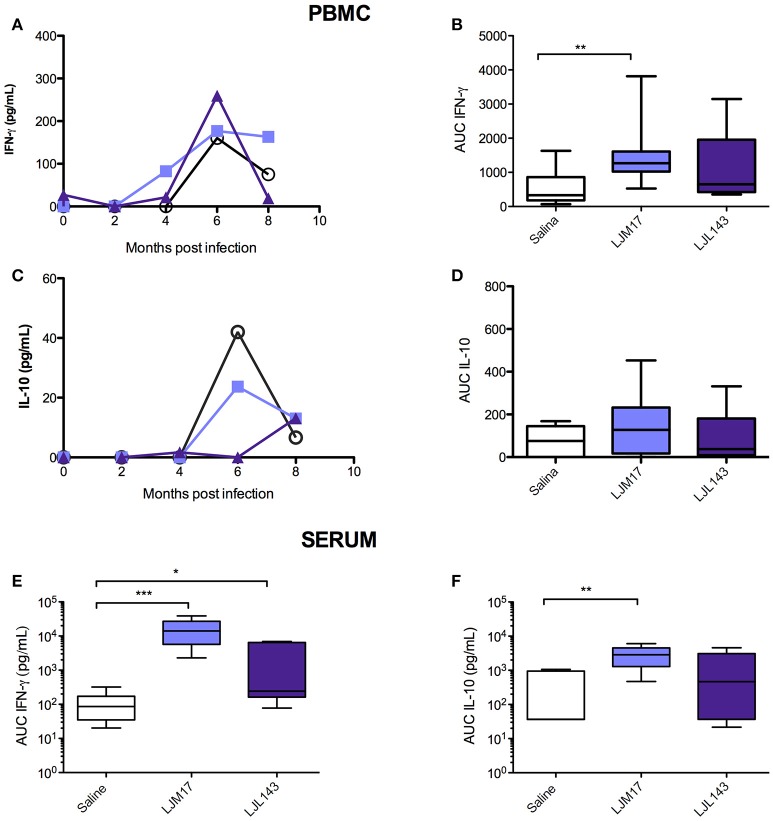
Detection of IFN-γ and IL-10 in PBMC culture supernatant and in sera from immunized dogs and controls challenged with *L. infantum*. Beagle dogs (*n* = 10 animals/group; total of 30 dogs) were immunized following vaccination strategies described in the methodology section. Thirty days after the final immunization, dogs were challenged with *L. infantum* in the presence of *L. longipalpis* saliva. PBMCs from immunized and control animals were collected at T0 (15 days after immunization), and every 2 months until 8 months after infection„ then stimulated with *L. infantum* (1:10); supernatants were collected after 24 (IL-10) and 48 (IFN-γ) hours and analyzed by ELISA to detect IFN-γ and IL-10. Sera from immunized and control animals were collected at T0, T1 and T2 to detect IFN-γ and IL-10 by the Luminex technique. **(A)** PBMC IFN-γ, **(B)** PBMC IFN-γ AUC **(C)** PBMC IL-10, **(D)** PBMC IL-10 AUC, **(D)** sera IFN- γ AUC **(E)** sera IL-10 AUC, **(F)** sera Luminex technique. For the statistical analysis, the area under curve was calculated, followed by the Kruskall-Wallis test with Dunns post-test (*P < 0.05, **P < 0.005, ***P < 0.0001).

By calculating the area under the curve for all time points evaluated (0–8 months post-challenge), we observed that the animals immunized with LJM17 presented significantly higher amounts of IFN-γ compared to the other analyzed groups (*P* < 0.0078) (Figures 3A,B;Supplementary Table 6). Regarding IL-10, no differences were observed between the immunized and control dogs, as demonstrated in Figures 3C,D. Interestingly, while IFN- γ was elevated in both canine sera and PBMCs (Figures 3A,B,E;Supplementary Tables 6,7), this was not the case with IL-10, as significantly increased amounts of this cytokine were only found in LJM17-immunized canine sera (*P* < 0.0027) (Figures 3C,D,F; Supplementary Tables 6, 7).

### Additional cytokines and chemokines detected in immunized dog sera following infection

Using the Luminex technique, we evaluated the production of different cytokines (IFN-γ, IL-10, IL-2, IL-6, IL-7, IL-15, IL-18, and TNF) and chemokines (IL-8, CXCL10, GM-CSF, CXCL1, CCL2) in the sera of immunized and infected animals, as well as infected controls at T0, T1 and T2. We measured the cytokines and chemokines at these time points because after 2 and 4 months after infection corresponding to the presence and absence of parasites in the skin and spleen as well as in the serology, respectively.

**Figure 4:**
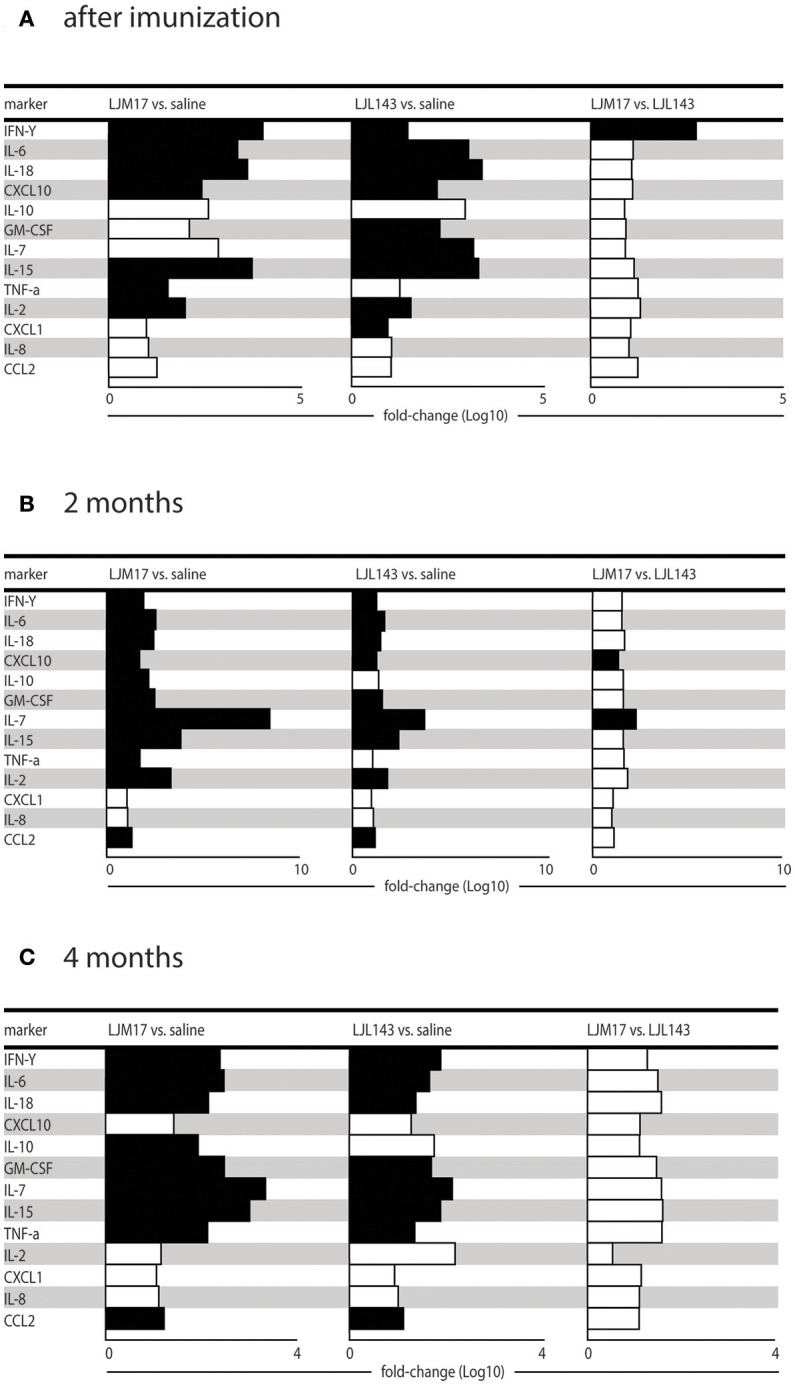
Cytokine and chemokine expression profiles in immunized dogs and controls. Beagle dogs (*n* = 10 animals/group; total of 30 dogs) were immunized following the vaccination strategies described in the methodology section. Thirty days after the final immunization, dogs were challenged with *L. infantum* in the presence of *L. longipalpis* saliva. Immunized and control animal sera were collected at T0 (15 days after immunization), T1 and T2 (2 and 4months after infection) to detect cytokines and chemokines in the sera. **(A)** Cytokines and chemokines 15 days after immunization, **(B)** cytokines and chemokines 2 months after infection and **(C)** cytokines and chemokines 4 months after infection.

At T0 (after immunization), the groups immunized with salivary proteins demonstrated an inflammatory immune response pattern with significantly increased IFN-γ, IL-6, IL-18, CXCL10, IL-15, and IL-2 in comparison to controls. The LJM17-immunized group also showed a significant increase in TNF production compared to controls, as well as in IFN-γ in comparison to the LJL143-immunized dogs. On the other hand, significant differences in GM-CSF, IL-7 and CXCL1 were seen only in the LJL143-immunized group as compared to controls. No differences were observed between the immunized groups and controls with regard to the production of IL-10 and the chemokines IL-8 and CCL2 (Figure 4A).

At T1, 2 months post-infection, the LM17- and LJL143-immunized dogs presented higher levels of IFN-γ, IL-6, IL-18, CXCL10, GM-CSF, IL-7, IL-15, and CCL2 in comparison to controls. However, only the LJM17-immunized animals had elevated IL-10 and TNF after *L. infantum* infection when compared to controls and the LJL143-immunized animals (Figure 4B). No significant differences were detected with respect to IL-8, CXCL1, and CCL2 chemokine production (Data not shown).

At 4 months after infection (T2), the dogs immunized with either salivary protein presented higher levels of IFN-γ, IL-6, IL-18, GM-CSF, IL-7, IL-15, TNF, and CCL2 when compared to controls. In addition, both experimental groups exhibited decreased CXCL10 and IL-2 in comparison to T1 (Figure 4C).

### Hierarchical cluster analysis

**Figure 5:**
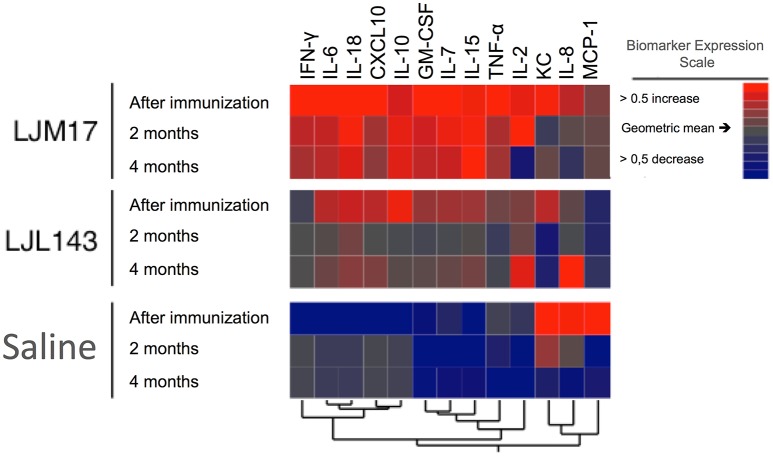
Cytokine and chemokine expression profiles in immunized dogs and controls. Beagle dogs (n = 10 animals/group; total of 30 dogs) were immunized following the vaccination strategies described in the methodology section. Thirty days after the final immunization, dogs were challenged with *L. infantum* in the presence of *L. longipalpis* saliva. Immunized and control animal sera were collected after immunization (T0) T1 and T2 (2 and 4 months after infection) to detect cytokines and chemokines. The hierarchical cluster analysis expression scale is representative of a relative change from the geometric mean of the entire study population (*n* = 30).

Based on data obtained from the Luminex assays, we performed a hierarchical cluster analysis to construct a heatmap to illustrate correlations among the analyzed cytokines and chemokines. The LJM17-immunized dogs presented a prolonged pattern of pro-inflammatory response that persisted until 4 months after infection, with the exception of IL-2, CXCL10, IL-8, and CCL2. Although immunization with LJL143 resulted in an immediate inflammatory response after immunization, this was markedly less intense. The expression of most of these molecules was lower at 2 and 4 months after infection (T1 and T2), suggesting that the LJL143 experimental immunization protocol did not induce a persistent response. As expected, control animals did not present any increases in the inflammatory molecules evaluated, even at T1 and T2 (Figure 5).

## Discussion

Previous studies have demonstrated that *L. longipalpis* salivary antigens (LJM17 and LJL143) are immunogenic in dogs, humans and foxes (13, 19), inducing both humoral and cellular Th1 immune responses in dogs, and leading to an immune profile consistent with immune mechanisms expected to effectively control CVL (14, 20). Collin et al. immunized dogs with these salivary proteins using a very complex immunization strategy, which resulted in systemic and local Th1 cell-mediated immunity that interfered with parasite survival *in vitro* (13). In light of these initial results, we simplified the immunization strategy using the same salivary antigens but with a less complex heterologous prime-boost vaccination strategy.

The experimental immunization strategy reported here also elicited a strong humoral and cellular immune response. Both LJM17 and LJL143-immunized dogs showed elevated production of specific IgG1 and IgG2, which indicates the induction of a mixed (Th1/Th2) immune response. In addition, the increased IFN-γ production observed in both stimulated PBMCs as well as in sera of immunized dogs, together with increases in other cytokines and chemokines suggests that the simplified evaluated prime-boost vaccination schedule provides immune responses qualitatively similar to those previously obtained by Collin et al. (13). These results are encouraging, since the prime-boost vaccination protocol will be more straightforward for future implementation in the field in endemic areas.

Many studies have found that cytokine production plays an essential role in the host inflammatory process, leading to the activation of lymphocytes and macrophages (20–23). In our chemokine analysis, we observed that all immunized dogs presented significantly higher levels of CXCL10 and GM-CSF compared to control dogs. These chemokines have been associated with inflammatory stimulation, which induces the differentiation of monocytes and granulocyte precursor cells (24, 25).

The analysis of the data obtained in the present study with immunized and infected dogs indicates that LJM17 antigen induced an outspoken immune response with higher levels of IFN-γ compared to IL-10, which is consistent with expected protective immune mechanisms against infection by *L. infantum*. Indeed, post-infection cytokine and chemokine production indicated higher levels of IFN-γ in PBMCs and sera for LJM17-immunized dogs in comparison to controls. Studies in experimentally infected dogs have demonstrated that protective immunity is associated with IFN-γ production, as well as with the activation of cytotoxic T cells (3, 14). Therefore, the present results stand in agreement with Collin et al., who demonstrated that animals vaccinated with LJM17 elicited a predominant Th1 immune response, as evidenced by the greater predominance of IFN-γ over IL-10, which may lead to a leishmanicidal effect via macrophage activation (13). Rafati et al. showed that high levels of IL-10 may be associated with increased parasite burden and disease progression (26). Indeed, many patients with VL produce high levels of IL-10 that could potentially inhibit the action of inflammatory cytokines, such as IFN-γ and TNF (14, 27). Additionally, it has already been shown that clinically ill animals with high parasitism demonstrate a predominant accumulation of IL-10 (28), further confirming that the IFN-γ/IL-10 ratio may be a relevant indicator of vaccine efficacy (26).

Other cytokines potentially indicative of protection in CVL were also evaluated in sera of immunized dogs before they were challenged with *L. infantum*. We found that infected dogs immunized with LJM17 presented higher levels of IL-2, IL-6, IL-7, IL-15, IL-18, and TNF upon infectious challenge, which suggests that these animals maintained a long-term immune profile consistent with expected protective mechanisms. The protective role of IL-2 and TNF has been extensively discussed in the literature, and the presence of these cytokines has been associated with resistance to *L. infantum* in experimentally and naturally infected dogs (20, 23, 29, 30). Increased levels of IL-2, TNF, and IFN-γ were detected in the sera of LJM17-immunized dogs, suggesting that immunization in the presence of this salivary protein has the potential to improve protection of dogs against *Leishmania* infection. In addition, several studies focused on the immune response to CVL have also demonstrated increases in IL-6 in both asymptomatic and uninfected dogs (15, 21, 29, 31).

Cytokines originating from myeloid cells, such as IL-15 and IL-18, have been associated with the activation of mature NK cells, which in turn produce IL-12 (32, 33). The LJM17-immunized dogs evaluated here also exhibited increased production of these cytokines, which further reinforces the potential of this approach for vaccine development.

All of the immunized infected animals presented higher levels of GMCSF and CXCL10 when compared to controls. The role of GMCSF has been widely studied, mainly in the context of infection by several *Leishmania* species (34, 35). The stimulatory effects exerted by this chemokine on monocytes and macrophages lead to the production of high levels of proinflammatory cytokines and chemokines, which intensifies the leishmanicidal effect of these cells (24) and activates cytotoxic NK cells, thus inducing IFN-γ production and the recruitment of Th1-activated cells (36). In addition, a recent study conducted by our group found that naturally and experimentally infected asymptomatic dogs that express GMCSF and CXCL10 presented low parasite loads and low clinical scores, suggesting that the upregulation of these two chemokines may represent biomarkers for controlled infections (37).

Due to the low infection pressure produced by our experimental model, all dogs became infected with *L. infantum* but none developed progressive CVL. This permitted the assessment of the immune response to the parasite in immunized compared to control dogs, but did not provide adequate experimental settings to assess whether the tested vaccine can limit the development of clinical disease, nor produce evidence of protection in the time period evaluated. Although the infection in our groups was mild, we were able to re-isolate the parasites, pointing out their viability. We also detected the presence of antibodies anti-Leishmania IgG, suggesting the establishment of infection. It is important to emphasize that in our study the serology anti-Leishmania, in fact, is one more indicator of infection and not protection against the parasite, since antibodies did not contribute to the protective immune response (3, 29). In experimental transmission, the route of administration of the infectious challenge inoculum can greatly influence the development of disease. Accordingly, intradermal, subcutaneous, intravenous, intraperitoneal, and intracardiac routes are often employed as inoculation routes for challenge strategies with *Leishmania* (38, 39, 40). However, these different routes may directly influence the development and the pathogenicity of infection (3). In natural transmission, each bite by a *L. infantum*-infected *L. longipalpis* sand fly can inject up to 10^4^ parasites into host skin, along with immunogenic molecules, including salivary proteins and promastigote secretory gel (41–43). To this end, sand fly-transmitted parasites reportedly enhance the severity of the infection (44–46). Additionally, despite the considerably lower amounts of parasites transmitted by natural infections as compared to experimental infection models, in the natural environment mammalian hosts can be extensively exposed to dozens of sand flies and subjected to successive bites. The present study employed a single intradermal challenge involving 10^7^ *L. infantum* promastigotes together with *L. longipalpis* saliva. Although the same model of experimental infection proved successful in the development of disease of variable severity in previously evaluated animals (14), it is important to note the existence of several barriers in the context of carrying out experimental infection, including genetic variability among experimental dogs, potential inefficiencies related to the intradermal route of infection, variable parasite virulence, the fact that the animals were well-nourished and received regular veterinary care, and the duration of the evaluation time period, among others. Collectively, all or some of these aspects may help to explain the limitations faced in our study, as illustrated by the lack of clinical disease development in infected dogs.

Nevertheless, assessment of the immune response in LJM17- and LJL143-immunized dogs after infection with *L. infantum* compared to the response of control dogs provided useful information. Our results indicated that LJM17 induces a stronger and longer lasting immunity than LJL143 when compared to control dogs. As such, this study places more emphasis on the LJM17 antigen over the LJL143 one for a future vaccine component. Of practical interest, it confirmed the immunogenicity of LJM17 following a simplified, field-applicable vaccination approach. However, protective efficacy of a vaccine containing the LJM17 antigen against CVL clinical disease remains to be further explored by exposure of immunized dogs to a natural infection by sand flies either experimentally or in an endemic area setting.

In summary, the present study found that animals immunized with the LJM17 salivary protein from *L. longipalpis* presented a sustained and predominantly Th1-oriented cellular immune response after experimental infection with *L. infantum*. This was evidenced by the detection of both relevant proinflammatory cytokines and chemokines. Further studies conducted in endemic areas will be necessary to more comprehensively evaluate the benefits of the inclusion of these salivary proteins in *Leishmania* vaccines to protect against clinical disease.

## Author contributions

Conceived and designed the experiments: MA, CT, and CB. Performed the experiments: MA, JC, VA, LdS, MB, MS, and CT. Analysed the data: MA, BA, LF, PV, SK, and CB. Contributed reagents, materials, analysis tools: JV, LF, LG-S, MSS, DMF, BA, CB, and PB. Wrote the paper: MA, PV, CB, and BA.

### Conflict of interest statement

The authors declare that the research was conducted in the absence of any commercial or financial relationships that could be construed as a potential conflict of interest.
