# Corrigendum: Immunization of Experimental Dogs With Salivary Proteins From *Lutzomyia longipalpis*, Using DNA and Recombinant Canarypox Virus Induces Immune Responses Consistent With Protection Against *Leishmania infantum*

**DOI:** 10.3389/fimmu.2019.01828

**Published:** 2019-08-12

**Authors:** Melissa Moura Costa Abbehusen, Jurema Cunha, Martha Sena Suarez, Clarissa Teixeira, Valter dos Anjos Almeida, Laís da Silva Pereira, Marcelo Bordoni, Leonardo Gil-Santana, Manuela da Silva Solcà, Deborah Bittencourt Moté Fraga, Laurent Fischer, Patricia Torres Bozza, Patricia Sampaio Tavares Veras, Jesus G. Valenzuela, Shaden Kamhawi, Bruno B. Andrade, Claudia I. Brodskyn

**Affiliations:** ^1^Fundação Oswaldo Cruz, Instituto Gonçalo Moniz, Salvador, Brazil; ^2^Fiocruz Piauí, Fundação Oswaldo Cruz, Teresina, Brazil; ^3^Boerhinger Ingelheim, R&D, Laboratoire de Lyon Portes des Alpes, Lyon, France; ^4^Laboratório de Imunofarmacologia, Fundação Oswaldo Cruz, Instituto Oswaldo Cruz, Rio de Janeiro, Brazil; ^5^Vector Molecular Biology Unit, Laboratory of Malaria and Vector Research, National Institute of Allergy and Infectious Diseases, National Institutes of Health, Bethesda, MD, United States; ^6^Multinational Organization Network Sponsoring Translational and Epidemiological Research (MONSTER) Initiative, Fundação José Silveira, Salvador, Brazil; ^7^Escola Bahiana de Medicina e Saúde Pública, Salvador, Brazil; ^8^Universidade Salvador (UNIFACS), Laureate Universities, Salvador, Brazil; ^9^Faculdade de Medicina and Instituto de Ciências da Saúde, Universidade Federal da Bahia, Salvador, Brazil; ^10^Nacional de Ciência e Tecnologia de Investigação em Imunologia (III-INCT), São Paulo, Brazil

**Keywords:** vaccine, sand fly, canine visceral leishmaniasis, disease vectors, salivary proteins

“Patricia Sampaio Tavares Veras” was not included as an author in the published article. The corrected **Author Contributions** statement appears below.

“Conceived and designed the experiments: MA, CT, and CB. Performed the experiments: MA, JC, VA, LdS, MB, MS, and CT. Analysed the data: MA, BA, LF, PV, SK, and CB. Contributed reagents, materials, analysis tools: JV, LF, LG-S, MSS, DMF, BA, CB, and PB. Wrote the paper: MA, PV, CB, and BA.”

The authors apologize for this error and state that this does not change the scientific conclusions of the article in any way. The original article has been updated.

