# Supplementary material for: Immunization of Experimental Dogs With Salivary Proteins From Lutzomyia longipalpis, Using DNA and Recombinant Canarypox Virus Induces Immune Responses Consistent With Protection Against Leishmania infantum
Source: Front Immunol. 2018 Nov 16;9:2558. doi: 10.3389/fimmu.2018.02558 (PMC6251279; doi:10.3389/fimmu.2018.02558)
Supplement: Supplementary file 1 [file Data_Sheet_1.PDF]

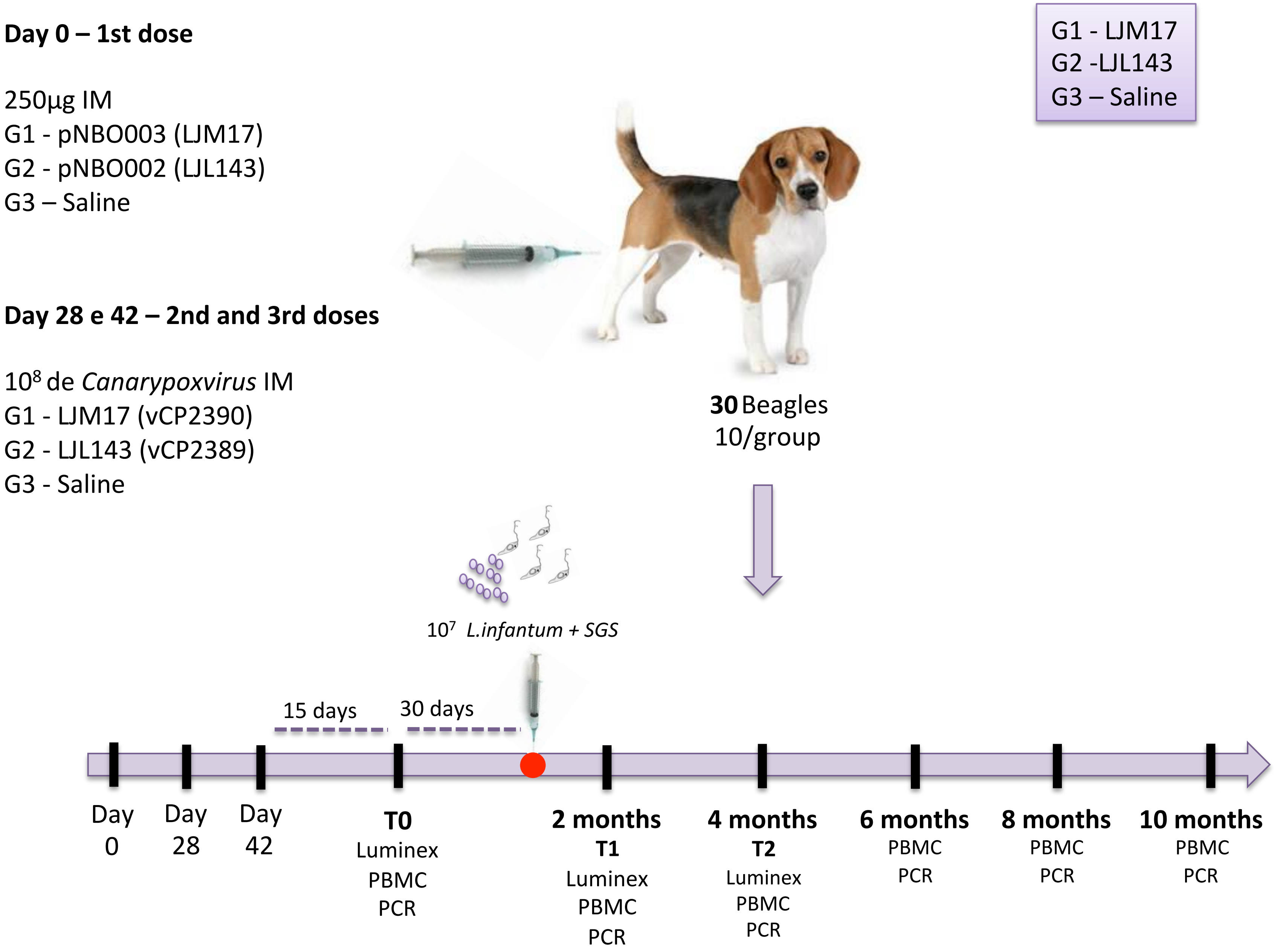
Day 0 – 1st dose
250µg IM
G1 - pNBO003 (LJM17)
G2 - pNBO002 (LJL143)
G3 – Saline
Day 28 e 42 – 2nd and 3rd doses
10^8 de *Canarypoxvirus* IM
G1 - LJM17 (vCP2390)
G2 - LJL143 (vCP2389)
G3 - Saline
G1 - LJM17
G2 -LJL143
G3 – Saline
30 Beagles
10/group
10^7 *L.infantum + SGS*
15 days
30 days
Day 0
Day 28
Day 42
T0
Luminex
PBMC
PCR
2 months
T1
Luminex
PBMC
PCR
4 months
T2
Luminex
PBMC
PCR
6 months
PBMC
PCR
8 months
PBMC
PCR
10 months
PBMC
PCR
