# Supplementary material for: Immunization of Experimental Dogs With Salivary Proteins From Lutzomyia longipalpis, Using DNA and Recombinant Canarypox Virus Induces Immune Responses Consistent With Protection Against Leishmania infantum
Source: Front Immunol. 2018 Nov 16;9:2558. doi: 10.3389/fimmu.2018.02558 (PMC6251279; doi:10.3389/fimmu.2018.02558)
Supplement: Supplementary file 2 [file Data_Sheet_2.PDF]

Supplementary Table 1 - Tabulated data of humoral immune response (total IgG anti-LJM17, anti-LJL143 and specific IgG1/IgG2 subclasses) in immunized and control dogs

Beagles ID	IgG anti-LJM17 (OD)*			IgG anti-LJL143 (OD)*			IgG1/IgG2 (OD)*		
	1	2	Mean	1	2	Mean	1	2	Mean
**Control group**									
**119598**	0,092	0,103	0,098	0,105	0,113	0,109	0,308	0,278	0,293
**119594**	0,095	0,093	0,094	0,104	0,101	0,103	0,210	0,203	0,207
**119593**	0,059	0,076	0,068	0,074	0,152	0,113	0,277	0,270	0,274
**119600**	0,079	0,073	0,076	0,086	0,130	0,108	0,306	0,306	0,306
**119592**	0,116	0,118	0,117	0,109	0,125	0,117	*0,905*	*0,875*	0,890
**113230**	0,055	0,062	0,059	0,080	0,075	0,078	0,037	0,031	0,034
**119591**	0,055	0,083	0,069	0,189	0,139	0,164	0,314	0,444	0,379
**113235**	0,046	0,044	0,045	0,145	0,122	0,134	0,236	0,208	0,222
**113238**	0,056	0,083	0,070	0,056	0,083	0,070	0,200	0,211	0,205
**113228**	0,118	0,119	0,119	0,317	0,154	0,236	0,281	0,290	0,286
**LJM17 immunized group**									
**113237**	1,228	1,301	1,265	0,337	0,303	0,320	0,856	0,847	0,851
**111541**	1,207	1,164	1,186	0,250	0,249	0,250	0,548	0,556	0,552
**113221**	1,338	1,298	1,318	0,267	0,284	0,276	*3,608*	*1,609*	2,609
**119595**	1,420	1,383	1,402	0,362	0,334	0,348	0,906	0,942	0,924
**113226**	1,509	1,456	1,483	0,402	0,390	0,396	0,986	0,955	0,970
**113224**	0,965	0,871	0,918	0,285	0,334	0,310	1,001	1,034	1,017
**113225**	1,522	1,496	1,509	0,428	0,460	0,444	0,653	0,636	0,644
**113334**	1,386	1,359	1,373	0,378	0,391	0,385	0,812	0,797	0,805
**113236**	1,400	1,270	1,335	0,253	0,285	0,269	0,648	0,671	0,660
**119597**	1,404	1,372	1,388	0,333	0,347	0,340	0,664	0,637	0,651
**LJL143 immunized group**									
**113222**	0,368	0,348	0,358	0,991	1,003	0,997	1,570	1,750	1,660
**113231**	0,379	0,350	0,365	1,202	1,238	1,220	1,403	1,427	1,415
**111545**	0,177	0,177	0,177	1,214	1,289	1,252	1,137	0,933	1,035
**113240**	0,238	0,201	0,220	1,115	1,325	1,220	0,817	0,995	0,906
**113229**	0,352	0,418	0,385	1,348	1,274	1,311	1,830	1,758	1,794
**111548**	0,258	0,275	0,267	1,194	1,200	1,197	1,944	1,773	1,858
**113233**	0,284	0,243	0,264	1,217	1,204	1,211	1,623	1,559	1,591
**113232**	0,299	0,299	0,299	1,163	1,235	1,199	0,654	0,799	0,727
**111547**	0,257	0,225	0,241	1,176	1,136	1,156	1,247	1,341	1,294
**111552**	0,370	0,328	0,349	1,150	1,124	1,137	0,545	0,495	0,520

*Samples were evaluated in duplicate (1, 2) and the mean OD was calculated
Representative Data from Figure 1 A, B and C, respectively
