# Supplementary material for: Immunization of Experimental Dogs With Salivary Proteins From Lutzomyia longipalpis, Using DNA and Recombinant Canarypox Virus Induces Immune Responses Consistent With Protection Against Leishmania infantum
Source: Front Immunol. 2018 Nov 16;9:2558. doi: 10.3389/fimmu.2018.02558 (PMC6251279; doi:10.3389/fimmu.2018.02558)
Supplement: Supplementary file 3 [file Data_Sheet_3.PDF]

Supplementary Table 2 - Tabulated data of Cellular immune response assessed by IFN- γ ELISA using supernatant of PBMCs stimulated by recombinant salivary proteins or SGH and by LUMINEX analysis of serum cytokines concentration (IFN- γ, IL-10 and IFN-γ/IL-10 ratio) of control, LJM17 immunized, LJL143 immunized animals after immunization protocol

Beagles ID	Mean OD of IFN- γ in the supernatant of PBMCs Stimulated by		Serum cytokines concentration (pg/mL)		
			IFN- γ	IL-10	IFN- γ / IL-10
**Control group***	**Culture Medium**	**SGH**			
**119598**	0,00	155,37	0,00	0,01	1,00
**119594**	0,00	6,15	0,00	0,01	1,00
**119593**	0,00	0,00	0,00	0,00	1,00
**119600**	0,00	0,00	173,67	1027,07	0,17
**119592**	177,15	0,00	0,00	0,01	1,00
**113230**	295,49	0,00	0,00	0,01	0,00
**119591**	0,00	0,00	0,00	977,76	0,00
**113235**	179,48	0,00	0,00	0,00	1,00
**113238**	0,00	0,00	0,00	724,58	0,00
**113228**	0,00	0,00	0,00	0,00	1,00
**LJM17 immunized group****	**rLJM17**	**SGH**			
**113237**	0,00	0,00	2058,08	0,00	0,71
**111541**	0,00	0,00	19302,51	2886,23	24,82
**113221**	300,06	234,79	2616,79	777,78	26167880,00
**119595**	0,00	0,00	4319,81	0,00	43198140,00
**113226**	0,00	0,00	16835,88	0,00	11,10
**113224**	375,62	1981,36	3720,42	1516,41	37204230,00
**113225**	37,52	0,00	1347,66	0,00	0,50
**113334**	0,00	37,78	16423,92	2685,91	164239200,00
**113236**	0,00	97,66	19787,09	0,00	19787090,00
**119597**	184,97	217,10	0,00	0,00	1,00
**LJL143 immunized group*****	**rLJL143**	**SGH**			
**113222**	297,08	241,73	31,85	0,00	318490,00
**113231**	0,00	0,00	7,55	0,00	7552,00
**111545**	54,31	0,00	0,00	0,00	0,00
**113240**	309,97	495,17	2695,03	984,10	2,11
**113229**	0,00	12,97	1121,02	1277,41	11210220,00
**111548**	0,00	53,32	0,00	0,00	0,00
**113233**	0,00	346,00	0,00	2070,24	0,10
**113232**	89,40	0,00	0,00	0,00	0,00
**111547**	208,19	344,55	4,95	789,49	0,02
**111552**	176,94	449,15	52,85	300,18	528520,00

* PBMCs stimulated by 4 µg/mL of culture medium and SGH of *L. longipalpis*
** PBMCs stimulated by 4 µg/mL of rLJM17 and SGH of *L. longipalpis*
*** PBMCs stimulated by 4 µg/mL of rLJL143, and SGH of *L. longipalpis*
Representative Data from Figure 1 D, E F and G, respectively
