# Supplementary material for: Immunization of Experimental Dogs With Salivary Proteins From Lutzomyia longipalpis, Using DNA and Recombinant Canarypox Virus Induces Immune Responses Consistent With Protection Against Leishmania infantum
Source: Front Immunol. 2018 Nov 16;9:2558. doi: 10.3389/fimmu.2018.02558 (PMC6251279; doi:10.3389/fimmu.2018.02558)
Supplement: Supplementary file 4 [file Data_Sheet_4.PDF]

Supplementary Table 3 – Tabulated data of humoral immune response (anti-*Leishmania* IgG antibodies), in canine sera from control, LJM17 and LJL143 immunized and *L. infantum*-infected groups, from infection challenge (T0) until 10 months after infection, assessed every 2 months

Beagles ID	Anti-*Leishmania* IgG antibodies mean OD in different months after infection challenge*					
	**T0**	**T2**	**T4**	**T6**	**T8**	**T10**
**Control group**						
**119598**	0,05	0,16	0,05	0,14	0,04	0,02
**119594**	0,09	0,20	0,06	0,20	0,08	0,07
**119593**	0,07	0,29	0,07	0,23	0,09	0,08
**119600**	0,06	0,27	0,05	0,14	0,05	0,07
**119592**	0,05	0,17	0,03	0,10	0,04	0,05
**113230**	0,03	0,18	0,03	0,09	0,06	0,03
**119591**	0,06	0,18	0,05	0,24	0,07	0,08
**113235**	0,07	0,24	0,05	0,11	0,07	0,06
**113238**	0,04	0,11	0,03	0,10	0,04	0,05
**113228**	0,11	0,51	0,05	0,17	0,15	0,04
**LJM17 immunized group**						
**113237**	0,05	0,39	0,13	0,28	0,19	0,29
**111541**	0,04	0,32	0,08	0,52	0,10	0,10
**113221**	0,04	0,19	0,07	0,22	0,11	0,07
**119595**	0,10	0,47	0,18	0,39	0,31	0,28
**113226**	0,04	0,25	0,08	0,45	0,14	0,05
**113224**	0,04	0,16	0,03	0,10	0,05	0,05
**113225**	0,03	0,19	0,07	0,34	0,11	0,10
**113334**	0,06	0,25	0,07	0,24	0,16	0,13
**113236**	0,07	0,29	0,08	0,19	0,09	0,12
**119597**	0,05	0,19	0,08	0,39	0,10	0,12
**LJL143 immunized group**						
**113222**	0,05	0,33	0,04	0,22	0,06	0,06
**113231**	0,03	0,23	0,08	0,19	0,14	0,13
**111545**	0,03	0,26	0,03	0,20	0,05	0,03
**113240**	0,02	0,22	0,07	0,20	0,09	0,15
**113229**	0,05	0,22	0,13	0,25	0,27	0,23
**111548**	0,05	0,18	0,04	0,14	0,07	0,06
**113233**	0,06	0,34	0,09	0,18	0,11	0,11
**113232**	0,06	0,17	0,05	0,14	0,07	0,13
**111547**	0,03	0,25	0,03	0,43	0,14	0,06
**111552**	0,05	0,28	0,07	0,20	0,16	0,12

*Mean OD of duplicate evaluation of each sample
Representative Data from Figure 2 A, B and C, respectively
