# Supplementary material for: Immunization of Experimental Dogs With Salivary Proteins From Lutzomyia longipalpis, Using DNA and Recombinant Canarypox Virus Induces Immune Responses Consistent With Protection Against Leishmania infantum
Source: Front Immunol. 2018 Nov 16;9:2558. doi: 10.3389/fimmu.2018.02558 (PMC6251279; doi:10.3389/fimmu.2018.02558)
Supplement: Supplementary file 5 [file Data_Sheet_5.PDF]

Supplementary Table 4 – Tabulated data of parasite loads (in spleen and skin samples) from each dog, after challenge infection two months after infection in control, LJM17 and LJL143 immunized and *L. infantum*-infected groups.

Beagles ID	Parasite Load	
	Spleen (parasite/ml)	Skin (parasite/mg)
**Control group**		
**119598**	1,00	5,00
**119594**	44,00	2,70
**119593**	3201,00	4,30
**119600**	1,00	1,10
**119592**	69,00	17,60
**113230**	4,00	4,00
**119591**	81,00	2,30
**113235**	3142,00	45,90
**113238**	16,00	4,30
**113228**	532,00	1,00
**LJM17 immunized group**		
**113237**	2,00	304,30
**111541**	702,00	1,00
**113221**	3,00	22,00
**119595**	155,00	98,10
**113226**	58,00	27,60
**113224**	1,00	1,00
**113225**	226,00	11,80
**113334**	879,00	1,00
**113236**	4,00	8,30
**119597**	173,00	7,00
**LJL143 immunized group**		
**113222**	1,00	48,90
**113231**	42,00	18,80
**111545**	23,00	60,50
**113240**	53,00	12,00
**113229**	7,00	3,60
**111548**	51,00	1,70
**113233**	28,00	53,20
**113232**	11,00	113,90
**111547**	4,00	6,40
**111552**	1,00	2,40

Representative Data from Figure 2D and 2E
