# Supplementary material for: Immunization of Experimental Dogs With Salivary Proteins From Lutzomyia longipalpis, Using DNA and Recombinant Canarypox Virus Induces Immune Responses Consistent With Protection Against Leishmania infantum
Source: Front Immunol. 2018 Nov 16;9:2558. doi: 10.3389/fimmu.2018.02558 (PMC6251279; doi:10.3389/fimmu.2018.02558)
Supplement: Supplementary file 6 [file Data_Sheet_6.PDF]

Supplementary Table 5 – Tabulated data of clinical evaluation scores and Area Under Curve (AUC) of the clinical scores from each dog, after challenge infection from T0 to 10 months, in control, LJM17 and LJL143 immunized and *L. infantum*-Infected groups

Beagles ID	Clinical evaluation scores in different months after infection challenge						Clinical score AUC
	T0	T2	T4	T6	T8	T10	
**Control group**							
**119598**	1	3	0	2	2	1	16
**119594**	1	1	1	0	0	2	7
**119593**	0	2	0	3	3	1	17
**119600**	2	2	1	0	1	1	11
**119592**	1	3	0	3	2	4	21
**113230**	2	1	0	3	6	0	22
**119591**	1	1	0	2	3	2	15
**113235**	1	1	1	2	2	2	15
**113238**	2	1	0	2	3	5	19
**113228**	4	2	1	1	2	5	21
**LJM17 immunized group**							
**113237**	2	2	0	6	5	5	33
**111541**	2	2	0	0	3	3	15
**113221**	1	0	0	0	2	3	8
**119595**	1	1	0	2	1	3	12
**113226**	0	1	0	0	2	1	7
**113224**	1	0	0	0	0	2	3
**113225**	1	1	0	1	3	2	13
**113334**	2	2	2	2	2	3	21
**113236**	1	1	1	1	1	4	13
**119597**	0	1	1	1	1	4	12
**LJL143 immunized group**							
**113222**	1	1	0	4	2	1	16
**113231**	1	1	0	0	3	3	12
**111545**	0	1	1	2	4	4	20
**113240**	0	0	0	1	3	1	9
**113229**	0	0	0	0	4	2	10
**111548**	1	1	0	3	1	3	14
**113233**	0	2	1	3	3	1	19
**113232**	2	1	2	0	2	5	17
**111547**	2	2	2	1	1	0	14
**111552**	2	0	1	1	2	4	14

Representative Data from Figure 2F and 2G, respectively
