# Supplementary material for: Immunization of Experimental Dogs With Salivary Proteins From Lutzomyia longipalpis, Using DNA and Recombinant Canarypox Virus Induces Immune Responses Consistent With Protection Against Leishmania infantum
Source: Front Immunol. 2018 Nov 16;9:2558. doi: 10.3389/fimmu.2018.02558 (PMC6251279; doi:10.3389/fimmu.2018.02558)
Supplement: Supplementary file 7 [file Data_Sheet_7.PDF]

Supplementary Table 6 – Tabulated data of cellular immune response assessed by IFN-γ and IL-10 ELISA using supernatant of PBMCs stimulated by *L. infantum*, and Area Under Curve (AUC) of IFN-γ and IL-10 ELISA results from each dog, after challenge infection from T0 to 8 months, in control, LJM17 and LJL143 immunized and *L.infantum*-infected groups

Beagles ID	Mean OD of IFN-γ and IL-10 in the supernatant of PBMCs stimulated by *L. infantum**										AUC	
	T0		T2		T4		T6		T8			
	IFN-γ	IL-10	IFN-γ	IL-10	IFN-γ	IL-10	IFN-γ	IL-10	IFN-γ	IL-10	IFN-γ	IL-10
**Control group**												
**119598**	0,0	0,0	0,0	0,0	0,0	0,0	141,7	54,7	0,0	44,8	283,5	154,2
**119594**	0,0	0,0	0,0	0,0	0,0	0,0	0,0	73,9	0,0	20,6	0,0	168,4
**119593**	0,0	0,0	0,0	0,0	0,0	0,0	0,0	69,2	70,1	22,2	70,1	69,2
**119600**	0,0	0,0	0,0	0,0	0,0	0,0	166,5	35,5	0,0	11,4	332,9	82,4
**119592**	177,2	0,0	0,0	0,0	0,0	0,0	0,0	48,5	80,2	44,9	80,2	141,8
**113230**	295,5	0,0	0,0	0,0	0,0	0,0	279,4	53,6	139,8	0,0	698,5	107,1
**119591**	0,0	0,0	0,0	0,0	0,0	0,0	774,8	0,0	83,0	1,9	1633,0	1,9
**113235**	179,5	0,0	0,0	0,0	0,0	0,0	153,6	0,0	0,0	0,0	307,1	0,0
**113238**	0,0	0,0	0,0	0,0	0,0	0,0	252,7	0,0	517,8	0,0	1023,0	0,0
**113228**	0,0	0,0	0,0	0,0	0,0	0,0	211,1	0,0	201,5	0,0	623,7	0,0
**LJM17 immunized group**												
**113237**	0,0	0,0	0,0	0,0	114,8	9,2	465,6	47,4	127,6	10,5	1288,0	123,8
**111541**	0,0	0,0	0,0	0,0	867,3	0,0	0,0	226,3	18,9	0,0	1754,0	452,7
**113221**	300,1	0,0	0,0	0,0	29,9	0,0	0,0	56,1	466,2	19,4	526,1	131,7
**119595**	0,0	0,0	0,0	0,0	261,9	2,2	0,0	111,6	413,0	0,0	936,8	227,8
**113226**	0,0	0,0	0,0	0,0	1366,3	0,1	541,2	115,1	0,0	15,6	3815,0	246,2
**113224**	375,6	0,0	0,0	0,0	389,0	0,0	246,2	0,0	0,0	137,3	1270,0	137,3
**113225**	37,5	0,0	0,0	0,0	50,1	0,0	404,3	0,0	198,8	0,0	1108,0	0,0
**113334**	0,0	0,0	0,0	0,0	0,0	1,8	107,6	0,0	922,0	18,8	1137,0	22,3
**113236**	0,0	0,0	0,0	0,0	0,0	0,0	632,4	0,0	201,6	45,7	1466,0	45,7
**119597**	185,0	0,0	0,0	0,0	0,0	0,0	0,0	0,0	22,3	0,0	22,3	0,0
**LJL143 immunized group**												
**113222**	0,0	0,0	0,0	0,0	487,0	2,0	625,8	85,5	21,0	26,6	2247,0	201,7
**113231**	0,0	0,0	0,0	0,0	0,0	5,4	0,0	0,0	0,0	0,0	0,0	10,9
**111545**	54,3	0,0	0,0	0,0	1573,6	4,4	0,0	0,0	17,1	34,0	3147,0	42,7
**113240**	310,0	0,0	0,0	0,0	89,9	0,0	414,7	85,2	143,3	3,9	1153,0	174,3
**113229**	0,0	0,0	0,0	0,0	43,0	0,0	0,0	52,0	269,9	21,5	356,0	125,6
**111548**	0,0	0,0	0,0	0,0	327,1	1,4	505,0	151,1	0,0	26,9	1664,0	331,9
**113233**	0,0	0,0	0,0	0,0	0,0	0,0	312,2	0,0	0,0	0,0	624,5	0,0
**113232**	89,4	0,0	0,0	0,0	0,0	2,0	274,3	0,0	104,8	0,0	653,4	4,0
**111547**	208,2	0,0	0,0	0,0	0,0	0,0	244,7	0,0	0,0	33,2	489,5	33,3
**111552**	176,9	0,0	0,0	0,0	0,0	3,2	0,0	0,0	351,6	4,7	351,6	11,1

*Mean OD of duplicate evaluation of each sample

Representative Data from Figure 3 A, B, C and D respectively
