# Supplementary material for: Immunization of Experimental Dogs With Salivary Proteins From Lutzomyia longipalpis, Using DNA and Recombinant Canarypox Virus Induces Immune Responses Consistent With Protection Against Leishmania infantum
Source: Front Immunol. 2018 Nov 16;9:2558. doi: 10.3389/fimmu.2018.02558 (PMC6251279; doi:10.3389/fimmu.2018.02558)
Supplement: Supplementary file 8 [file Data_Sheet_8.PDF]

Supplementary Table 7 – Tabulated data of and Area Under Curve (AUC) of LUMINEX results of serum cytokines concentration (IFN-γ and IL-10) from each dog, after challenge infection from T0 to 8 months, in control, LJM17 and LJL143 immunized and *L.infantum*-infected groups

Beagles ID	AUC	
	IFN-γ	IL-10
**Control group**		
**119598**	101,80	36,63
**119594**	94,87	36,63
**119593**	20,20	36,62
**119600**	319,80	1064,00
**119592**	24,41	36,63
**113230**	0,00	0,00
**119591**	77,25	1014,00
**113235**	194,90	36,62
**113238**	66,98	761,20
**113228**	0	0
**LJM17 immunized group**		
**113237**	2294,00	6033,00
**111541**	0,00	1200,00
**113221**	38879,00	0,00
**119595**	5479,00	1538,00
**113226**	18628,00	3175,00
**113224**	6392,00	469,70
**113225**	9570,00	4192,00
**113334**	27080,00	4673,00
**113236**	26911,00	2523,00
**119597**	20,37	0
**LJL143 immunized group**		
**113222**	232,50	36,62
**113231**	0,00	0,00
**111545**	77,60	1010,00
**113240**	6889,00	3074,00
**113229**	1988,00	262,30
**111548**	6460,00	4580,00
**113233**	0,00	0,00
**113232**	0,00	0,00
**111547**	244,00	468,40
**111552**	163,10	21,58

Representative Data from Figure 3E and 3F respectively
